# Cross-species gene enrichment revealed a single population of Hilsa shad (*Tenualosa ilisha*) with low genetic variation in Bangladesh waters

**DOI:** 10.1038/s41598-021-90864-6

**Published:** 2021-06-02

**Authors:** Anirban Sarker, Junlong Jiang, Habibon Naher, Junman Huang, Kishor Kumar Sarker, Guoxing Yin, Mohammad Abdul Baki, Chenhong Li

**Affiliations:** 1 Shanghai Universities Key Laboratory of Marine Animal Taxonomy and Evolution, Shanghai Ocean University, Shanghai, 201306 China; 2 Department of Zoology, Jagannath University, Dhaka, 1100 Bangladesh

**Keywords:** Ecology, Ecological genetics

## Abstract

*Tenualosa ilisha* is a popular anadromous and significant trans-boundary fish. For sustainable management and conservation of this fish, drawing an appropriate picture reflecting population status of this species is very essential based on their all-strategic habitats in Bangladesh. In this study, 139 samples from 18 sites were collected and cross-species gene enrichment method was applied. Like most of the Clupeiforms, nucleotide diversity of this shad was very low (0.001245–0.006612). Population differences between most of the locations were low and not significant (*P* > 0.05). However, *P* values of a few locations were significant (*P* < 0.05) but their pairwise F_ST_ values were very poor (0.0042–0.0993), which is inadequate to recognize any local populations. Our study revealed that the presence of a single population in the Bangladesh waters with some admixtured individuals, which may contain partial genes from other populations. Most of the individuals were admixed without showing any precise grouping in the ML IQtree and Network, which might due to their highly migratory nature. Fishes from haors and small coastal rivers were not unique and no genetic differences between migratory cohorts. The hilsa shad fishery should be managed considering it as a single panmictic population in Bangladesh with low genetic diversity.

## Introduction

**Figure 1:**
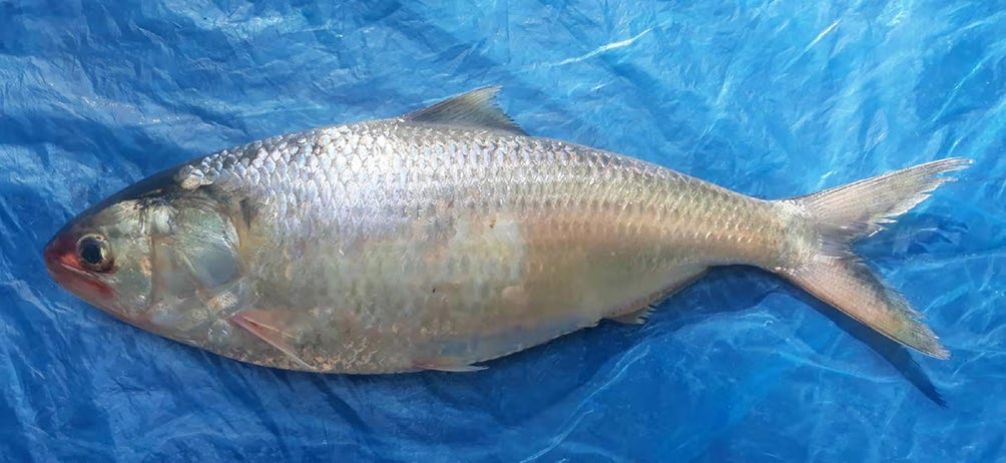
A Hilsa shad (*Tenualosa Ilisha)* specimen collected from Chandpur (CP), Bangladesh.

The national fish of Bangladesh, the Hilsa shad (*Tenualosa ilisha* Hamilton, 1822) (Family: Clupeidae) (Fig. 1) is a well-liked anadromous fish species^1^, locally known as Ilish. This is a significant trans-boundary fish, which survives in the Bay of Bengal and migrates between marine and freshwater for breeding, nursing and feeding purposes. Bangladesh enjoys major share of this migrating fish from the Bay of Bengal to upstream rivers (86%), followed by India (8%), and Myanmar (4%)^2^. The species is also distributed in Iran, Iraq, Saudi Arabia, Kuwait, Qatar, Oman, UAE, Pakistan, Sri Lanka, Thailand, Malaysia and Vietnam^3^. It is the largest single species fishery contributing 44% of total catch in Bangladesh that accounts virtually about 12.09% of total fish production of the country, representing around 1% of the total gross domestic product of the country with annual catch of 517,189 metric tons (inland catch: 232,698 MT and Marine catch: 284,500 MT)^4^. Hilsa shad also remains a subsistence food for many poor coastal communities and a valuable resource for millions of people in the Bay of Bengal and its major associated river systems.

The Hilsa shad lives in shallow coastal waters of the Bay of Bengal and its estuaries but in the breeding season they migrates to the upstream rivers from the Bay of Bengal using lower part of the Meghna River. The lower part of the Meghna River is directly connected to the Meghna (upstream), the Padma (lower stream of the Ganges) and the Jamuna (New Bhrahmaputra River) rivers, established the main migratory route for this fish. At the same time, they use some other small coastal rivers like the Payra, the Bishkhali, the Balaswar, the Pashur etc., which are far away from the main migration route as their freshwater entry. The Pashur River passes through the world largest mangrove forest, Sunderban and connected with the Padma River at Hardinge Bridge through the Madhumoti and the Gorai River; establish another important route of Hilsa shad migration. The Kocha River mainly connected to the Bishkhali and the Balaswar River, which passes by the side of the mangrove forest^5,6^. Simultaneously, this fish uses some marshy wetland ecosystems known as haor and hill stream rivers (e.g., Someshwari River) situated in the northeastern part of Bangladesh for their migration. The water qualities and ecological factors of these habitats are different from the Hilsa’s other migration routes. Moreover, the south eastern coast of the Bay of Bengal also represent some oceanographic and water qualitative unique characteristics as a fish (e.g., Hilsa shad) habitat which is distinctly different from other portion of the bay^7^. These types of special small coastal rivers, river passes through the mangrove forest and wetlands were overlooked in previous population studies of the Hilsa shad. Thus, comprehensive scenario of Hilsa population is incomplete because of ignoring all small unique unfocused water bodies. Therefore, the present study was designed to consider all types of habitat including all primary and secondary routes of migration from the Bay of Bengal.

The Hilsa shad shows a range of movement patterns. Two periods of migration of this fish occur in a year, the first -one is correlated with the monsoon rain (June–October), which causes flood and the second-one happens in the general rise of temperature of the water in the estuaries after the close of winter (January-March)^8,9^. Hilsa shad shows some seasonal morphometric variations between winter and monsoon runs at Hoogly River estuary, India^10^. The temporal stability of Hilsa shad in Bangladesh water was figured out based on spawning, fecundity and sex ratio^8^, but how closely are those are reflected in genetic makeup, of each stock is needed to be tested using molecular data.

Migration is an important factor for drawing the fish population with high migratory potential. Some cases this nature prevent to make local population or cluster and often make admixtured individuals that obscure population differentiation^11^. Differentiation of anadromous fish population by local adaptation is challenging because of their elevated rate of gene flow, highly connected divergent population and large population size^12^. As a highly migratory species, Hilsa shad have less chance to create high population differentiation in its migratory routes. Bangladesh is a small riverine country and its most the water bodies are connected to each other by a diverse river network. This type of habitat claims precise checking of the presence of population differentiation for a highly migratory fish species (e.g., Hilsa shad).

Discovery of population genetic structure and genetic diversity are very important for sustainable ocean fisheries, which can provide basic information for the fisheries resource assessment and management^13^. Due to environmental changes, pollution and over exploitation, many fishes are losing genetic diversity currently^14^. Previous studies on genetic population structure of *T. ilisha* were mostly based on allozymes, allele frequencies, microsatellite DNA markers and mitochondrial DNA regions: Cytochrome b (CytB), ATPase 6&8 (ATPase), 12 s and 16 s rRNA^10,15–18^. Mitochondrial gene analysis indicated temporal stability of sampled populations; with low genetic differentiation between temporal samples from same locality of three Indian rivers^10^. However, these methods bases on single or few loci, which have lesser power to estimate fine genetic structure. Very recently, A study discover the population genomics and structure of Hilsa shad in Bangladesh waters based on six and eight locations respectively using NextRAD sequencing^19,20^. But their studied locations did not represent overall water bodies of Bangladesh, like lentic water (haor), hill stream river (e.g., Someshwari river) and small coastal rivers (i.e., Pashur river, Kocha river, Lata Chapli river and Tetulia river) including the middle portion of Bangladesh (e.g., Manikganj, Bhairab etc.) and south eastern portion of the Bay of Bengal. According to these studies, Hilsa population was divided into two genetically structured clusters, marine and estuarine and fresh water, based on their local adaptation. Finally, the riverine population was divided into north-western riverine (turbid freshwater) and the north-eastern riverine (clear fresh water) clusters. They drawn several specific populations or clusters in the upper streams of Bangladesh, however, this shad is not found in the freshwater all over the year round. All of the spawning grounds of this shad were discovered in the lower stretches of the Meghna River and some other small coastal rivers situated almost in the same area^6,21^. Moreover, as a highly migratory species, Hilsa shad population should not able to make different clusters in their migratory routes. In the present study, sufficient number of samples were collected from almost all tactical water ecosystems including all major rivers of Bangladesh (i.e., the Padma, the Meghna, the Jamuna and the Brahmaputra river), coastal waters of the Bay of Bengal (western and eastern) and its estuary (in total 18 locations). Locations also included previously unfocused lentic water (haor), hill stream river and small coastal rivers including the middle portion of Bangladesh. Moreover, samples were collected from seasonal migratory (i.e., summer vs. winter) cohorts. Our assumption is that, in order to draw the total picture of population of the Hilsa shad, sampling should be focused on diverse habitat types including all strategic ecosystems and migratory cohorts instead of different river sections of the same major drainages.

Therefore, to avoid these previously discussed confusions and better understanding, we collected sequence data of 4434 nuclear genes from 139 Hilsa samples taken from the Bay of Bengal, its estuaries and all possible lotic and lentic waters and two migratory cohorts, applying a cross-species gene enrichment method^22^, to examine the genetic diversity and population structure of this shad. Our goal is to provide a solid estimation of the population status of Hilsa shad using genome-wide data and to infer its genetic diversity. Our study will provide a comprehensible look into the genetic diversity of this commercially important species and an evaluation of its population genetic structure. The findings should be important for the management and conservation of this important fisheries resource.

## Material and methodology

### Sample collection and DNA extraction

**Figure 2:**
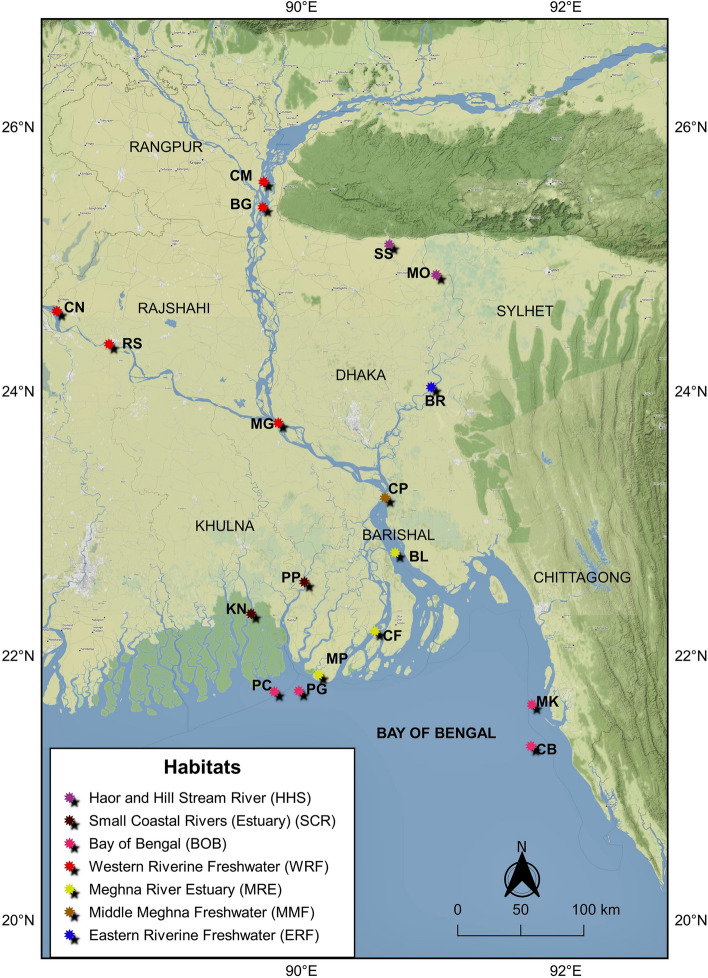
Map of sample collection sites of the highly migratory Hilsa shad across its diverse migratory habitats including all strategic aquatic ecosystems in Bangladesh. Each color indicates specific habitat group and abbreviated letter indicates sampling location. CM, Chilmari; BG, Balashi Ghat; CN, Chapai-Nababganj; RS, Rajshahi; MO, Mohanganj; SS, Someshwari river, Durgapur; MG, Manikganj; BR, Bhairab; CP, Chandpur ; KN, Khulna; PP, Pirojpur; BL, Bhola; MP, Mohipur; CF, Char Fasson; PC, Pokhkhir Char; PG, Patharghata; CB, Cox’s Bazar; MK, Maheshkhali. The map was produced by using QGIS version-3.4.1 with GRASS 7.4.2 (https://qgis.org/en/docs/index.html).

Samples (dead fish) have been taken from the commercial fishing boats or directly from fishermen at fish landing sites. In total 139 individuals of Hilsa shad were collected from the diverse ecosystems of Bangladesh including 18 locations involved all fresh water, brackish and marine habitats for this fish (Fig. 2). Furthermore, three primary routes of migration of Hilsa shad from Bay of Bengal were also considered. These sampling locations were categorized into seven different habitat groups based on their habitat nature i.e., 1. Western Riverine (Freshwater) 2. Eastern Riverine (Freshwater) 3. Haor and hill stream river 4. Middle Meghna 5. Meghna Estuary 6. Small Coastal Rivers (Estuary) 7. Bay of Bengal (Fig. 2, Table 1). The samples were identified based on morphological features^23,24^. Five closely related Kelee shad (*Hilsa kelee)* were collected from the Arabian Sea coast for using as out-group. For sampling, muscles were collected from the base of dorsal fin and fin clips were collected from the tip of caudal fin. For fixation and preservation of tissue samples, 100% and 95% ethanol were used respectively. Finally, samples were stored in 4^0^C refrigerator until DNA extraction started. DNA was extracted from 25 mg of tissue using an Ezup DNA extraction kit following the protocol of the manufacturer (Sangon Biotech, Shanghai, China).

**Table 1:** Sampling localities, number of samples from each site and sampling dates.

No	Habitat	Sample ID	Voucher number	Sampling Location	Water system	No. of samples	Latitude	Longitude	Sampling time
1	Western Riverine (Freshwater)	CL1364	SOU1801035-1 to 8	Chilmari (CM)	Brahmaputra River	8	25° 35′ 03.5ʺ N	89° 42′ 47.1ʺ E	Sep, 2017
		CL1350	SOU1801036-1to 6	Balashi Ghat (BG)	Jamuna River	6	25° 23′ 32.4ʺ N	89° 42′ 24.2ʺ E	Sep, 2016
		CL1348	SOU1801034-1 to 7	Chapai-Nababganj (CN)	Upper Padma River	7	24° 36′ 23.7ʺ N	88° 08′ 52.5ʺ E	Sep, 2016
		CL1349	SOU1801037-1 to 8	Rajshahi (RS)	Upper Padma River	8	24° 21′ 25.4ʺ N	88° 32′ 33.3ʺ E	Sep, 2016
		CL 1365	SOU1801038-1 to 10	Manikganj (MG)	Lower Padma River	10	23° 45′ 40.1ʺ N	89° 49′ 25.3ʺ E	Sep, 2017
2	Eastern Riverine (Freshwater)	CL 1363	SOU1801042-1 to 10	Bhairab (BR)	Upper Meghna River	10	24° 01′ 57.2ʺ N	90° 58′ 51.8ʺ E	Aug,2017
3	Haor and hill stream river	CL2317	SOU1801040-1 to 2	Mohanganj (MO)	Dingapota Haor	2	24° 52′ 50.3ʺ N	91° 01′ 10.2ʺ E	Aug, 2019
		CL2318	SOU1801041-1	Someshwari, Durgapur (SS)	Someshwari River	1	25° 06′ 31.4ʺ N	90° 39′ 47.7ʺ E	Aug, 2019
4	Middle Meghna	CL2026	SOU1801039-1 to 10	Chandpur (CP)	Lower Meghna River	10	23° 11′ 42.7ʺ N	90° 37′ 55.5ʺ E	Sep, 2018
5	Meghna Estuary	CL2036	SOU1801046-1 to 10	Bhola (BL)	Meghna River Estuary	10	22° 46′ 43.7ʺ N	90° 42′ 27.0ʺE	Sep, 2018
		CL 1356	SOU1801044-1 to 9	Mohipur (MP)	Lata Chapli River	9	21° 51′ 24.4ʺ N	90° 07′ 38.3ʺ E	Sep, 2016
		CL 1361	SOU1801048-1 to 8	Char Fasson (CF)	Tetulia River	8	22° 11′ 12.1ʺ N	90° 33′ 15.5ʺ E	Feb, 2017
6	Small Coastal Rivers (Estuary)	CL 2002	SOU1801043-1 to 8	Khulna (KN)	Pashur River	8	22° 19′ 05.0ʺ N	89° 37′ 04.4ʺ E	Sep, 2017
		CL 1355	SOU1801047-1 to 9	Pirojpur (PP)	Kocha River	9	22° 33′ 26.7ʺ N	90° 01′ 16.3ʺ E	Sep 2016
7	Bay of Bengal	CL 1359	SOU1801045-1 to 7	Pokhkhir Char (PC)	Bay of Bengal	7	21° 43′ 40.0ʺ N	89° 47′ 38.6ʺ E	Sep, 2016
		CL 1357	SOU1801049-1 to 10	Patharghata (PG)	Bay of Bengal	10	21° 43′ 58.4ʺ N	89° 58′ 46.1ʺ E	Oct, 2016
		CL2042	SOU1801051-1 to 9	Cox’s Bazar (CB)	Bay of Bengal	9	21° 18′ 55.6ʺ N	91° 44′ 09.4ʺ E	Sep, 2018
		CL 1360	SOU1801050-1 to 7	Maheshkhali (MK)	Bay of Bengal	7	21° 37′ 46.8ʺ N	91° 44′ 35.2ʺ E	Oct, 2016

### DNA library preparation, gene capture and sequencing

Extracted genomic DNA was sheared to about 500 bp using Covaris M220 Focused-ultrasonicator (Woburn, Massachusetts, USA) according to the manufacturer’ instructions. Size of sheared DNA and product of every further step was measured by using agarose gel electrophoresis. DNA libraries were constructed and “with-beads” method was adopted in this protocol to obtain higher yield^22^. Inline indices were added to the adapter to label the samples in the ligation step of library preparation to ease the possible risk of cross contamination among the samples during subsequent gene capture step. After that library preparation, products were pooled together equimolarly.

A cross species gene capture was done and genes were captured for two consecutive trials that increase the recovery rate of enriched gene^22^. A bait set was designed based on the sequence of two Clupeiform species *Denticeps clupeoides* (Acession number: GCA_900700345.2) and *Ilisha elongata* (unpublished) for capturing highest number of genes. The enriched libraries (average concentration: 17,073 ng/ml) were amplified by IS4 and indexing primers^25^. Finally, captured genes were pooled in equimolar ratios for sequencing on Illumina HiSeq X10 lane at Annoroad Inc (Beijing, China).

### Data preparation read assembly and post assembly processing

According to the description in Assexon pipeline^26^, data processing, read assembly and post assembly processing were done. Raw reads from each sample were parsed according to their 8 bp barcodes (139 unique barcodes were used) on P7 adapter using bcl2fastq v1.8.3 (Illumina). Trim galore v0.4.1 (http://www.bioinformatics.babraham.ac.uk/projects/trim_galore/) was used to trim low quality bases and sequence adaptors. Coding frame of each marker sequence was predicted and corrected using a Perl script (predict_frames.pl). Coding sequences were extracted and translated into amino acid sequences by using Bio:: Seq module in Bioperl^27^.

PCR duplicates were excluded by using “-fastx_uniques” command in USEARCH v10.0.240^28^. Sequence of *Danio rerio* (https://doi.org/10.5061/dyrad.2j5b4) was used as a reference sequence to parse reads to each gene file. Reads were sorted to references with BLAST hit using UBLAST with a relaxed e-value of 1 × 10^–4^. Reads of each locus were assembled (De novo assembly) into contigs by a conservative assembler SGA^29^. Contigs were locally aligned to protein sequences of references using the “protein2dna” model in the package under Exonerate^30^. Reciprocal blast method was used to pick up the orthologous genes.

The output amino acid (AA) sequences were aligned in batch using MAFFTv7.369b^31^. The AA sequences were translated back to DNA after alignment. Poor and badly aligned sequences in coding regions were removed by filter.pl to avoid interfere in phylogenetic inference. Summary statistics (e.g., number of enriched samples, GC content and percentage of missing data) for coding and flanking region of each locus and sample was extracted by using statistics.pl^26^.

### SNP calling

A custom Perl script was used to make consensus sequences for each target locus from assembled contigs^26^ and then reads with adapter sequences were trimmed (Quality Phred score cutoff: 20) and low quality reads were excluded and finally mapped to the consensus by using BWA v0.7.5. The sequence map format (SAM) files were converted into binary format (BAM) by using Samtools^32^. SNP sites were genotyped based on the BAM files using GATK-3.2.2^33^. GATK Best Practices recommendations were followed^34^. Single SNP per locus with least amount of missing data and highest quality score was kept for most analyses to meet the requirement of linkage equilibrium. Custom Perl script was used again to convert the SNP VCF file into NEXUS file and STRUCTURE input file^26^.

### Phylogenetic analysis and Network based on gene-capture data

A concatenated maximum likelihood (ML) tree was reconstructed under IQtree v1.6.9 with 1,000 bootstrap replicates using the aligned DNA sequences^35^. FigTree.v1.4.4 was used to visualize the consequential phylogenetic tree (http://tree.bio.ed.ac.uk/software/figtree/). Model selection and data partition were also automatically done by IQtree v1.6.9**.** A dendrogram based on the F_ST_ values was also made as implemented in the R package.

Network 5.0.1.1^36^was used to visualize genetic clustering of the individuals of different populations by making a median-joining network. To build the network, only 842 polymorphic sites were used. VCF2RDF converter was used to convert SNP VCF to .rdf file for using as an input file in Network analysis.

### Genetic variation analysis

SNP vcf file was converted into arlequin (.arp) file using PGDspider 2.1.1.2 with the input of population summary^37^. SNP arlequin (.arp) file was used as an input file in the analysis of molecular variance (AMOVA), which was performed using ARLEQUIN 3.5.2 with 10,000 permutations^38^. Nucleotide diversity for each population was computed by using DnaSP v6.12.03^39^. The SNP data of variant call format (.vcf) was used as an input file in DnaSP. Pairwise F_ST_ matrix^40^ supported by ARLEQUIN 3.5.2 was used to calculate the genetic variation among groups, among populations within groups and within populations. Text editor Notepad + was used to edit the project file at the time of AMOVA and F_ST_ analysis. Deviation from Hardy–Weinberg equilibrium for every location was calculated by ARLEQUIN. 3.5.2. Exact test used a Markov chain (for all Loci) where forecasted chain length: 1,000,000 and Dememorization steps: 100,000.

### Population clustering

Genetic partitioning of the 139 individuals was assessed using STRUCTURE v2.3.4^41^ based on the data containing only one SNP per locus. Initial burn was set in 50,000 replicates, followed by 500,000 replicates for each K (number of genetic clusters) for the STRUCTURE runs. The analysis were run for K = 1 − 18 (1 − total number of locations), each replicated three times. Best K was identified by STRUCTURE HARVESTER 0.693^42^. Finally, result of STRUCTURE with best K was transferred to plot form. The Ordination of the SNP genotypes was investigated using discriminant analysis of principal components (DAPC) was computed as implemented in the ADEGENT package^43^ and SNP VCF files were used as an input file.

### Animal research and ethics approval

Species used in this study (Hilsa shad: *Tenualosa ilisha*) is a food fish in Bangladesh. No live fish was used in this study. Samples (dead fish) were taking from commercial fishing boats or directly from fisherman at fish landing. Fish tissue sampling and protocols were approved by the ‘Ethics Committee for the Use of Animal Subjects’ of Shanghai Ocean University.

## Result

### Sequencing results (NGS)

Each sample produced 4,015,989 raw reads on average and then 4,000,188 filtered reads (on average) were obtained from raw reads after trimming off adapter sequences and reads with low quality score (Q < 20). After removing the 230,829 reads (on average) of PCR duplicates, 93.87% of filtered reads were scrutinized as unique reads (3,769,359 on average). From each sample 1,395 target loci were obtained on average with the best one had 2,223 loci and the lowest one had 504 captured loci (Table S1). The average number of captured loci of the out-group sample was 1,104. All loci (3,399 loci) of studied species and out-group were checked manually. Loci with weird segments, samples from only one location or with lower than four samples were also excluded. After exclusions, 2,461 loci were kept. The deviated locus number was (average): 1,344 (28.05%) and highest deviation from Hardy–Weinberg equilibrium was 0.00053.

### Phylogenetic relationships based on genome-scale nuclear data

**Figure 4:**
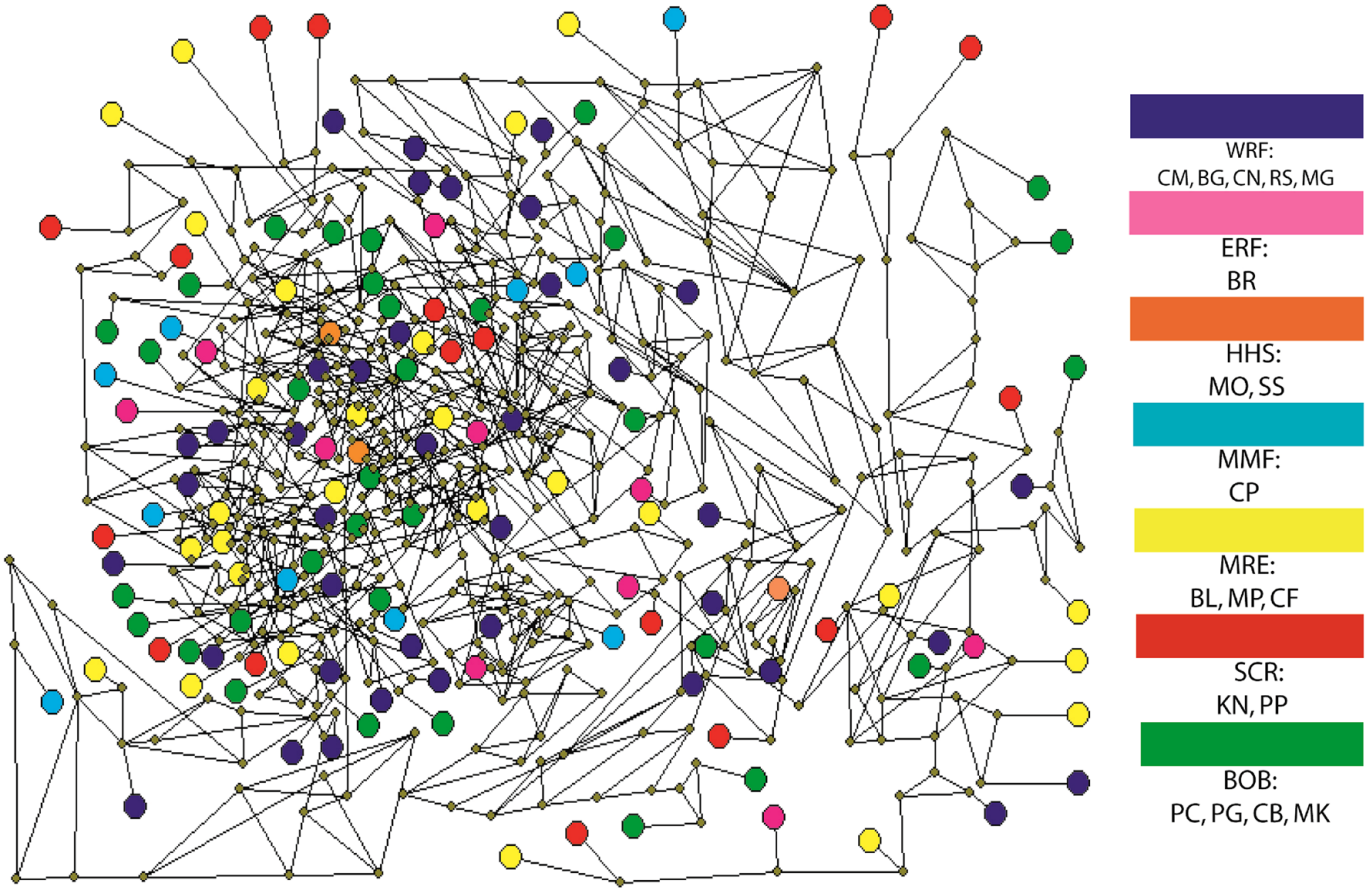
A median joining network of studied individuals based on 842 SNP loci by Network 5.0.1.1 (https://www.fluxus-engineering.com/sharenet_rn.htm). Each color indicates specific habitat group and abbreviated letter indicates sampling location. CM, Chilmari; BG, Balashi Ghat; CN, Chapai-Nababganj; RS, Rajshahi; MO, Mohanganj; SS, Someshwari, Durgapur; MG, Manikganj; BR, Bhairab; CP, Chandpur ; KN, Khulna; PP, Pirojpur; BL, Bhola; MP, Mohipur; CF, Char Fasson; PC, Pokhkhir Char; PG, Patharghata; CB, Cox’s Bazar; MK, Maheshkhali.

The maximum likelihood tree was built using IQtree on all of the individuals of Bangladesh waters, collected from different ecosystems mixed together. No location had any unique cluster, but some portion of phylogenetic tree had partial groupings (Fig. 3, S3). The phylogenetic tree revealed that the Hilsa shad represent a single genetic population in the Bangladesh water and there is no significant cluster. In network of 842 SNP loci, all samples were randomly interconnected together without any type of pattern (Fig. 4). There is no isolation based on distance, water quality, nature of the habitat and migratory seasons. Negligible samples of same location showed inter-connections among them. That means network result also supported the presence of single population in Bangladesh water like the assumption depicted from maximum likelihood IQtree.

**F﻿igure 3:**
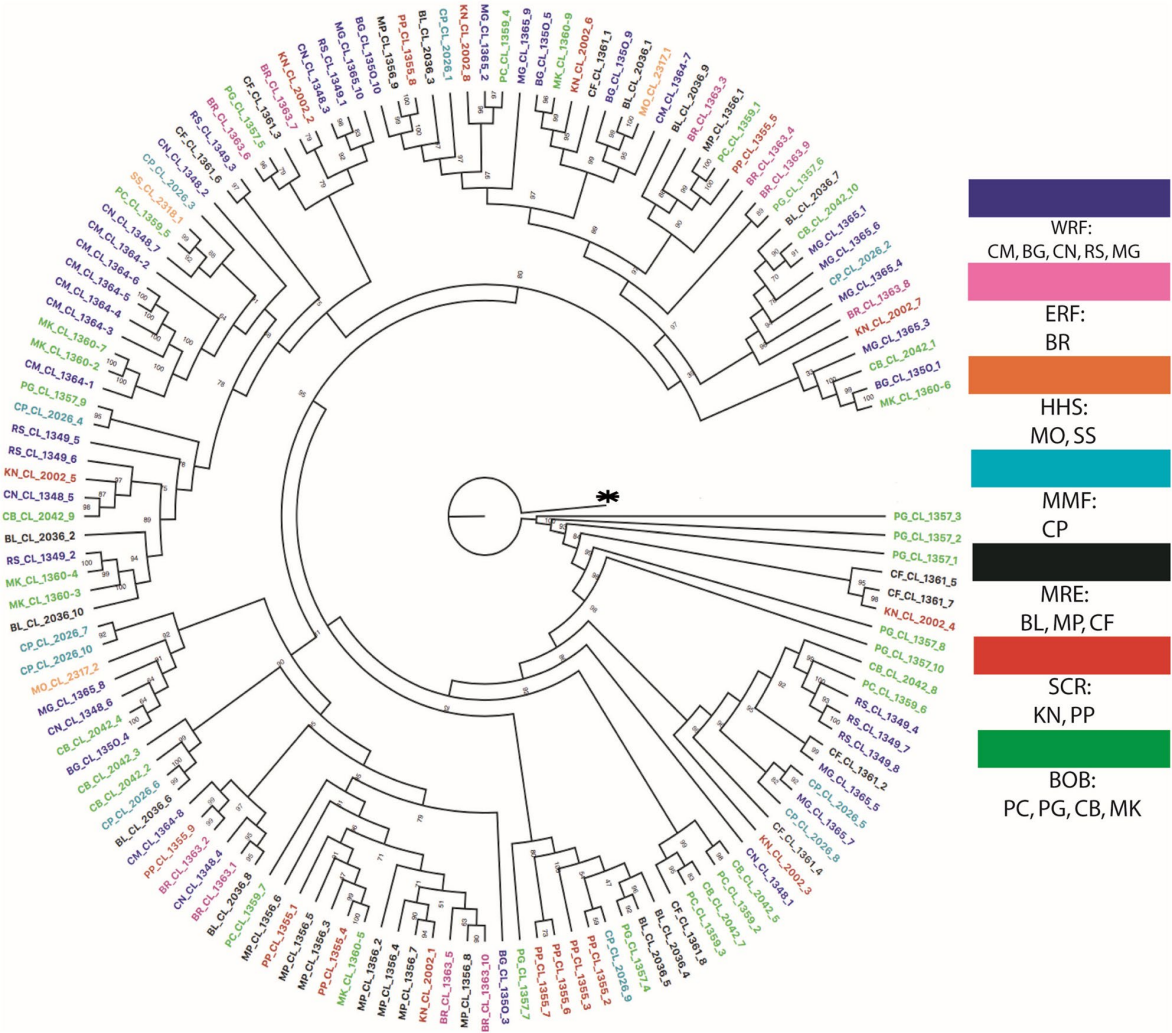
A ML tree based on sequences data concatenating 2,461 loci reconstructed by using IQtree v1.6.9 (http://www.iqtree.org/) with 1,000 bootstrap replicates. Each color indicates specific habitat group and abbreviated letter indicates sampling location (Table 1). CM, Chilmari; BG, Balashi Ghat; CN, Chapai-Nababganj; RS, Rajshahi; MO, Mohanganj; SS, Someshwari, Durgapur; MG, Manikganj; BR, Bhairab; CP, Chandpur ; KN, Khulna; PP, Pirojpur; BL, Bhola; MP, Mohipur; CF, Char Fasson; PC, Pokhkhir Char; PG, Patharghata; CB, Cox’s Bazar; MK, Maheshkhali.

### Genetic diversity and differentiation

Average nucleotide diversity (Pi) of Hilsa shad of Bangladesh waters was 0.004632, with highest value in Chilmari (0.008811) and lowest in Balashi Ghat (0.001809) (Table 2). Analysis of molecular variance (AMOVA) represented that percentage of variation among suspected significant habitat groups ( i.e., Western riverine freshwater, Eastern riverine freshwater, haor and hill stream river, Middle Meghna freshwater, Meghna river estuary, small coastal rivers, the Bay of Bengal) was very low (0.99%). Percentage of variations of among population within groups and within populations were 1.07% and 97.93% respectively (Table 3). Pairwise F_ST_ values of maximum locations were very poor (61% F_ST_ value was in between 0.0009 and 0.0993, 2% F_ST_ value was more than that value and rest showed negative value) and most of the case, *P* value was not significant. Populations of fresh water rivers (Western turbid and eastern clean rivers) also had poor F_ST_ value and non significant *P* value except between Manik Ganj (MG) and Chilmari (CM), which was more than significant level (*P* < 0.05) (Table 4). Populations of main migratory route and alternative migratory route had some differences. Samples of the Kocha river (PP) of alternative migratory route were different from all of the locations of main migratory route in the downstream (i.e., CF, MP, BL and CP) based on significant *P* value (*P* < 0.05) and samples of another alternative route location (KN) was also different from MP in the same way. However, F_ST_ values among them were not high. The dendrogram based on the F_ST_ values also represented same pattern (Fig. S4).

**Table 2:** Results of analysis of molecular variance (AMOVA).

Source of variation	d.f	Sum of squares	Variance components	Percentage of variation
Among groups	6	336	0.3669 Va	0.99
Among populations within groups	11	464	0.3965 Vb	1.07
Within populations	260	9411	36.1970 Vc	97.93
Total	277	10,211	36.9604

**Table 3:** Nucleotide diversity (Pi) of each population.

Code	Population	Theta (per site) from Pi
CM	Chilmari	0.008811
BG	Balashi Ghat	0.001809
CN	Chapai-Nababganj	0.005465
RS	Rajshahi	0.002277
SS	Someshwari, Durgapur	0.003821
MG	Manikganj	0.003508
BR	Bhairab	0.005068
CP	Chandpur	0.006612
KN	Khulna	0.001245
PP	Pirojpur	0.004889
BL	Bhola	0.005580
MP	Mohipur	0.003514
CF	Char Fasson	0.004328
PC	Pokhkhir Char	0.004756
PG	Patharghata	0.004543
CB	Cox’s Bazar	0.006501
MK	Maheshkhali	0.006025
Average		0.004632

**Table 4:** Pairwise differences (F_ST_) among populations.

	_**BG**_	_**CM**_	_**CN**_	_**RS**_	_**BR**_	_**BL**_	_**CF**_	_**MP**_	_**CB**_	_**MK**_	_**PC**_	_**PG**_	_**KN**_	_**PP**_	_**MG**_	_**MO**_	_**SS**_	_**CP**_
_BG_	_0.0000_																	
_CM_	_0.0000_	_0.0000_																
_CN_	_0.0179_	_0.0000_	_0.0000_															
_RS_	_0.0009_	_0.0035_	_0.0000_	_0.0000_														
_BR_	_0.0000_	_0.0036_	_0.0000_	_0.0000_	_0.0000_													
_BL_	_0.0178_	_0.0346*_	_0.0446*_	_0.0278*_	_0.0104_	_0.0000_												
_CF_	_0.0000_	_0.0000_	_0.0000_	_0.0000_	_0.0000_	_0.0225*_	_0.0000_											
_MP_	_0.0620*_	_0.0969*_	_0.0993*_	_0.0867*_	_0.0581*_	_0.0002_	_0.0736*_	_0.0000_										
_CB_	_0.0026_	_0.0047*_	_0.0000_	_0.0000_	_0.0000_	_0.0299*_	_0.0000_	_0.0799*_	_0.0000_									
_MK_	_0.0549_	_0.0434*_	_0.0567*_	_0.0756*_	_0.0253*_	_0.0128_	_0.0419*_	_0.0179*_	_0.0278*_	_0.0000_								
_PC_	_0.0000_	_0.0046_	_0.0000_	_0.0000_	_0.0000_	_0.0176*_	_0.0000_	_0.0742*_	_0.0000_	_0.0309_	_0.0000_							
_PG_	_0.0000_	_0.0135*_	_0.0000_	_0.0000_	_0.0000_	_0.0320*_	_0.0000_	_0.0745*_	_0.0000_	_0.0262*_	_0.0000_	_0.0000_						
_KN_	_0.0059_	_0.0235_	_0.0193_	_0.0211_	_0.0000_	_0.0027_	_0.0061_	_0.0359*_	_0.0162_	_0.0127_	_0.0127_	_0.0023_	_0.0000_					
_PP_	_0.0257*_	_0.0782*_	_0.0588*_	_0.0545*_	_0.0350*_	_0.0042*_	_0.0457*_	_0.0085*_	_0.0409*_	_0.0000_	_0.0351*_	_0.0514*_	_0.0131_	_0.0000_				
_MG_	_0.0111_	_0.0073*_	_0.0000_	_0.0000_	_0.0000_	_0.0466*_	_0.0000_	_0.1129*_	_0.0000_	_0.0704*_	_0.0000_	_0.0050_	_0.0359*_	_0.0709*_	_0.0000_			
_MO_	_0.1123_	_0.0081_	_0.0000_	_0.0673_	_0.0025_	_0.0360*_	_0.0423_	_0.1164*_	_0.0013_	_0.1043*_	_0.0180_	_0.0085_	_0.0730_	_0.0624*_	_0.0242*_	_0.0000_		
_SS_	_0.0572_	_0.0000_	_0.0000_	_0.0530_	_0.0000_	_0.0000_	_0.0000_	_0.0538_	_0.0000_	_0.0865_	_0.0000_	_0.0000_	_0.0281_	_0.0162_	_0.0000_	_0.0000_	_0.0000_	
_CP_	_0.0000_	_0.0000_	_0.0000_	_0.0000_	_0.0000_	_0.0106*_	_0.0000_	_0.0643*_	_0.0000_	_0.0344*_	_0.0000_	_0.0000_	_0.0004_	_0.0427*_	_0.0000_	_0.0254_	_0.0000_	_0.0000_

For abbreviations of population names, see Table 1, *Indicates significant *P* values (*P* < 0.05).

### Population structure

**Figure 5:**
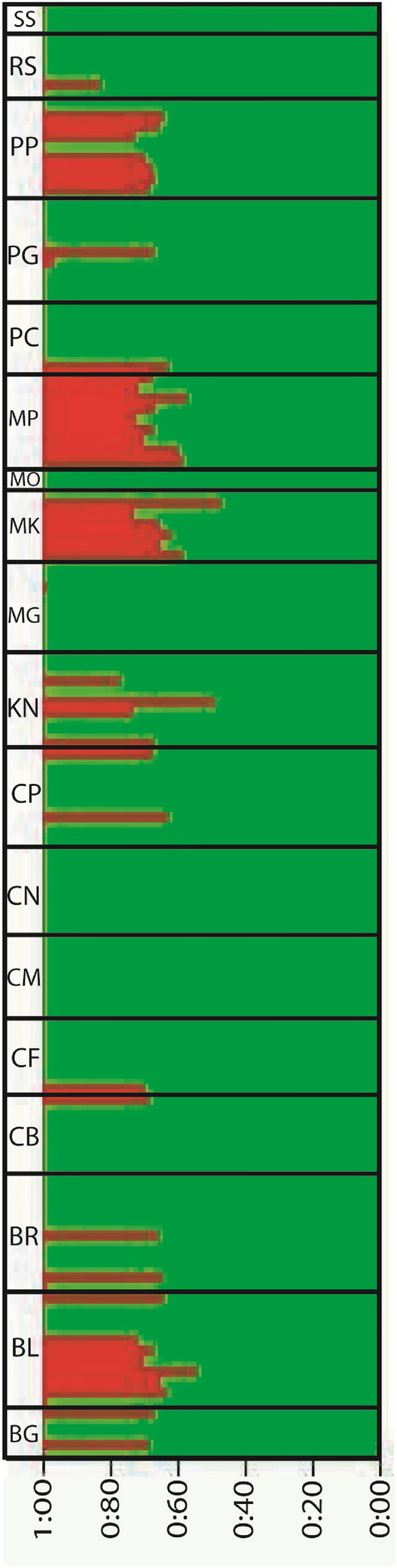
Structure analysis based on 842 SNP loci. CM, Chilmari; BG, Balashi Ghat; CN, Chapai-Nababganj; RS, Rajshahi; MO, Mohanganj; SS, Someshwari, Durgapur; MG, Manikganj; BR, Bhairab; CP, Chandpur ; KN, Khulna; PP, Pirojpur; BL, Bhola; MP, Mohipur; CF, Char Fasson; PC, Pokhkhir Char; PG, Patharghata; CB, Cox’s Bazar; MK, Maheshkhali.

Population of Hilsa shad belonged two groups (K = 2) was supported by Structure analysis, dominant group (green colored group) belonged to the maximum individuals of the population and only few individuals carrying some genes of other group (red colored group) along with dominant group genes (Fig. 5, Fig. S2, Table S2). Samples of CM, CN, MG, MO and SS belonged to the dominant group without any admixtured individuals whereas BG, BR, CB, CF, CP, PC, PG and RS mostly belonged to the dominant group with few admixtured individuals. Moreover, BL, KN, MK, MP and PP had more admixtured individuals than dominant group individuals (Fig. 5). There was no single location that had only admixtured individuals or no one individual that only carried the genes of other small group (red colored group). DAPC result was also similar to structure result (Fig. S1). All samples of Bangladesh water including all types of strategic ecosystems made a single cluster that represent only one population. There was no isolation between sea, estuary and freshwater ecosystems and no separate clusters between western and eastern freshwater rivers.

## Discussion

Present results showed that Hilsa shad had low nucleotide diversity (0.001809–0.008811) like most of the Clupeiforms, e.g., Elongate ilisha (0.001–0.010), Tapertail anchovy (0.0011–0.0029) in Yangtze river and Japanese anchovy (0.0014–0.0090)^44–46^. Sea fish population had higher genetic diversity than anadromous population within same species or among same group^47^. Although, Hilsa and Kelee shad belonged to the same subfamily Dorosomantinae but Hilsa shad is anadromous in nature and Kelee shad is exclusively marine^48^. Because of this habit, nucleotide diversity of Hilsa shad was lower than Kelee shad (*Hilsa kelee*) (0.010337–0.014690)^49^. Correspondingly, marine Pacific herring (0.020)^50^ also had higher nucleotide diversity than Hilsa shad. There were several researchers also reported low nucleotide diversity of Hilsa shad population in the Hoogli, the Ganges and the Brahmaputra river of India^10,17,18^. Low genetic diversity suggested that only small portion of the total population had the scope of successful spawning. That might be associated with their long anadromous breeding migration journey. At that time huge numbers of individuals were caught in their long migratory routes by the fishermen. Frequent changing of spawning pattern is another reason of unsuccessful spawning^51^. Therefore, Government of Bangladesh should place some safety and protection actions including, public conscious, restriction on fishing gear, Hilsa fisheries management activities and proper timing of the fishing ban period.

Previous studies on genetic population structure of *T. ilisha* were mostly based on allozymes, allele frequencies, microsatellite DNA markers and mitochondrial DNA regions: Cytochrome b (CytB), ATPase 6&8 (ATPase), 12 s and 16 s rRNA^10,15–18^. However, genomic data is more powerful marker than previous markers to present the history, evolution, population status and phylogeny of a fish. Recently, A study discover the population genomics and structure of Hilsa shad in Bangladesh waters based on genomic data at NGS platform by NextRAD sequencing, however they mistakenly assigned samples collected from the confluent of the Meghna River as the north-eastern riverine group^19,20^. Our study was also based on genomic data at the NGS platform. Conversely, we collected sequence data of 4434 nuclear genes applying a cross-species gene enrichment method^22^, to examine the genetic diversity and population status of hilsa shad from the Bay of Bengal, its estuaries and all possible lotic and lentic waters and two migratory cohorts.. This study provided a solid estimation of the population status of Hilsa shad using genome-wide data and to infer its genetic diversity.

Result of the maximum likelihood IQtree and the population structure suggested that the fresh, estuarine and marine water of Bangladesh have a single population of Hilsa shad. In-addition DAPC, dendrogram and network on SNP loci analysis also represented the same trend. In the phylogenetic tree, samples of all locations were mixed together without making any specific cluster. In the population structure analysis, a single population was present with some admixtured individuals bearing small portion of genes from other group. Pairwise F_ST_ value between most locations were poor with non-significant *P* value (*P* > 0.05), that support the deprived local population differences and homogeneity of this fish population throughout our studied locations. The hilsa shad population in Bangladesh might retrieve from a collapsed population. Once upon a time (upto first half of 1990s), this fish was most available and cheap fish in Bangladesh. Because of overexploitation and lack of proper management, the fish population was collapsed more than one decade. After that period, because of fishing ban period and public consciousness (first imposed in 2011), the population started to increase. Hilsa fish production in Bangladesh has doubled in a decade from 2006–2007 (279,189 MT) to 2017–2018 (517,189 MT)^4,64^. This fact probably caused low genetic diversity and divergence among populations of hilsa shad in the Bangladesh waters.

Bangladesh has diversified fresh water habitats for Hilsa shad migration including main river system, coastal and freshwater small rivers, hill stream rivers, haors etc. but anadromous migration of this shad starts from same marine water body, the Bay of Bengal, which is their living ground. Furthermore, this fish has highly migratory nature among marine, estuarine and fresh water bodies. Therefore, it is difficult to draw a conclusion that there is more than one population in this water system. Low variation among groups and among population within groups also did not support more than one population. F_ST_ value between most of the locations was poor with non-significant *P* value, which suggested that the population differences were not significant. Although in some cases, *P* value was significant but due to their poor F_ST_ value that did not provide strong support of local population differences. Here present findings of this study were supported by the findings of some previous researchers who represented the single gene pool or stock of this species in the Bay of Bengal with a substantial gene flow^18,52,53^.

All of the spawning grounds of Hilsa shad were identified in the coastal areas of Bangladesh especially at the lower stretches of the Meghna, the Tetulia, the Ander Manik and the Shahabazpur River e.g., Hatia (Moulavir char) Sandwip (Kalir char) and Bhola (Dhal char and Monpura)^6,21^. However, migratory plan is mainly initiated during the spawning season, which is activated with follow of fresh water runoff from the inland rivers, and naturally it occurs with the commencement of the south-west monsoon and consequent flooding of all the major rivers draining down to the upper Bay of Bengal and there are no considerable differences in any context. Isolation of spawning ground is an important factor for population differentiation^11^. Due to presence of un-alienated spawning grounds, it is less feasible to draw population differences of Hilsa shad in the upper streams of different rivers and in their living ground, Bay of Bengal. Therefore, the unique spawning grounds and sole major migratory down-stream route strengthen the presence of single population in all over the Bangladesh water without any significant population clusters. Without specify exact spawning grounds for every cluster, it is unrealistic to draw several clusters in this population.

Hilsa population studies in Indian part across the Hoogli, the Bhagirathi, the Ganges and the Brahmaputra Rivers also suggested single and genetically homogeneous population in Indian part^10,17,18^. Hilsa shad population of the Hoogli-Bhagirathi river system and Hilsa stock of Bangladesh water used same natal habitat, Bay of Bengal. Moreover, the River Ganges is the upstream of the Padma River (Bangladesh) and the Bhagirathi River (India) as well as the Brahmaputra is the upstream of the Jamuna River (Bangladesh). Most of the Hilsa shad of River Ganges comes from the Padma River and as the same way the Brahmaputra river has no other significant source of this fish except the Jamuna River. So genetic homogeneity and unique population across these rivers of Indian part also supported the Hilsa shad’s single population in the Bangladesh water.

Nevertheless, Rahman and Naevdal (2000) based on allozymes and muscle proteins as well as Mazumder and Alam (2009) based on mitochondrial D-loop region figured out more than one Hilsa population in Bangladesh waters^15,54^. Rahman and Naevdal (2000) mentioned two populations: 1. Marine and 2. Estuary and fresh water but they processed without explaining how this highly migratory species was separated into two distinct cohorts. Mazumder and Alam (2009) divided the population into two clusters like previous study but poor pairwise F_ST_ value between two groups showed that there were no differences between fresh water and marine-estuarine locations. Recently Asaduzzaman et al*.* (2020) reported three clusters in the Hilsa population in Bangladesh waters, first one was in marine and estuarine waters and another two belonged to north–western riverine (turbid freshwater) and north-eastern riverine (clear freshwater) ecotypes^20^. Existing of a single population, the most likely assumption from the present research varied with their findings. Our result suggested that as a highly migratory species, Hilsa shad is incapable to belong to more than one population when sampled at different sections of their migration route. Our postulation is the presence of single cluster in the Bangladesh water because all water bodies are almost connected to each other, raising high rate of gene flow and created large population size. Western and eastern river systems of Bangladesh have immaterial dissimilar water quality (e.g., turbidity) but this is not enough to make population differences of Hilsa shad since they migrate and start their life from same spawning grounds and used almost same route across the lower stream and coastal estuaries during their breeding migration. Asaduzzaman et al*.* (2020) reported that samples of the Meghna river (MR) was included in the north-eastern riverine (clear freshwater) ecotypes by DAPC and neighbor-joining tree analysis^20^. However, their sample collection site (MR) was located in the common migratory route for north–western riverine (turbid freshwater) and north-eastern riverine (clear freshwater) ecotypes. Therefore, this site should be representing the samples of both ecotypes rather than specific one.

If we draw several specific populations or clusters in the upper streams of Bangladesh that means we had the scope to find this shad in the freshwater all over the year round. However, in the freshwater of Bangladesh, this fish was available in the summer (June–October) and winter season (January-March) only; these were related to their summer and winter migration respectably^55^. If one or two groups of this fish, continue their complete lifecycle in the freshwater (Western/Eastern part of Bangladesh) that states the assurance of continuous supply of this fish almost year round. However, the original scenario does not support this hypothesis. Finally we can conclude that, only one population of this fish inhabit in the Bangladesh waters without any instance of different populations and clusters (2–4) but in some specific locations, they had some particular characteristics. The Bay of Bengal is their main living ground, at the time of their breeding they come to the freshwater upper streams, spawn in the estuaries and finally return to the sea. Therefore, using all the same ecosystems (sea, estuary and freshwater rivers) in a cyclic fashion is essential to support their life cycle, which certainly pushes all the individuals to belong a unique population.

In the population structure analysis, only one population of Hilsa shad was identified with some admixtured individuals (32%) containing partial genes from other population in the water bodies of Bangladesh. The mentioned other population might not represent the Hilsa population of the Hoogly and Bhagirathi river system, India because, the Hilsa shads of both migratory routes of Bangladesh and India showed genetic homogeneity^10,17^. The Ganges and Brahmaputra rivers of Indian part are the upstream of the Padma and the Jamuna river of Bangladesh and might be belonged to the same population. However, Hilsa population of the Arabian Sea was genetically heterogeneous from the Bay of Bengal^18^ and those different population genes of admixtured individuals might come from the Arabian Sea by oceanographic dispersion. Once (almost 18,000 years ago) the Arabian Sea had a close connection with the Bay of Bengal through the Laccadive Sea, the Gulf of Mannar and the Palk Bay. Therefore, this likely was an easy way for oceanographic dispersion of Hilsa shad between these two water bodies. After that period, a bridge of limestone shoals, coral reefs and tombolo called as ‘Ram Bridge’ or ‘Adam’s Bridge’ (about 48 km) originated between Pamban Island off the south-eastern coast of Tamil Nadu, India, and Mannar Island, off the north-western coast of Sri Lanka ^56,57^. Sarker et al*.* (2020) also mentioned that type of oceanographic dispersion between these two water bodies for another Clupeid fish species, *Hilsa kelee*^49^. The Irrawaddy, the Naaf and the Sittang River of Myanmar were also regarded as another important route for Hilsa migration^6,58^. There is also a possibility of inflowing of these different genes of other population from such population. Still there is no population structure study was conducted in the Myanmar part. Therefore, there is no scope to compare those admixtured individuals with the Hilsa population of Myanmar. However, for completing the full scenario, the Hilsa population of Myanmar also claims research attention in population genomics field.

In the present study, Samples of both migration cohorts (summer and winter) were studied. The maximum likelihood IQ tree, population structure and DAPC suggested that samples of both migration cohorts were homogenous. Similarly, Jhingran and Natarajan (1969) and Ramakrishnaiah (1972) also did not find any significant temporal population differences in their previous studies^59,60^. Dwivedi (2019) found morphometric variations between seasonal migrants of Hilsa shad from Hooghly estuary, India using geometric morphometrics (GM) data^61^. They explained that these morphotypes might be related to the food availability and temperature fluctuation of summer and winter season but they did not incorporate that to the genetic level of the population. Quddus et al. (1984) reported two seasonal migratory populations of Hilsa shad in Bangladesh water based on spawning, fecundity and sex ratio^8^. Based on our findings and previous studies we can conclude these mentioned seasonal cohorts might be associated with their food availability and breeding rather than genome level.

Hill stream river and haor were two important and unique ecosystems for fish diversity in Bangladesh, they belong to the unique characteristics in the ecological factors as well as fish diversity^62,63^. Infrequently Hilsa shad use these two water bodies as their migratory routes. Samples were collected from the Shomeswari River and the Dingapota Haor, Mohanganj as the representatives of hill stream river and haor population respectively. However, Hilsa shad of these two exclusive water bodies were similar to the samples of the some other fresh water bodies (i.e., CM, CN and MG) as they were belonging to the Hilsa population without any admixtured individuals. Samples of SS do not have any significant *P* value with other locations whereas MO samples had significant *P* value with five other locations but having poor F_ST_ value with three locations (i.e., BL, PP, MG). MO samples had only mentionable F_ST_ value with MP (estuarine) and MK (marine), which might be the result of differences in water quality of these two water bodies. In DAPC, phylogenetic tree and in network, the samples of hill stream river and haor failed to make any unique cluster or monophyletic clade that represent they are also the part of single unique Hilsa population of Bangladesh waters.

Main migration was occurred through the Meghna river estuary, which is connected to the Padma, Meghna and Jamuna river system. However, there are some other alternative routes through some small coastal rivers e.g., the Pashur, the Bishkhali, the Balaswar, the Kocha river, which are connected to the Padma river through the Modhumati and the Gorai river. These coastal rivers passed through or beside the world largest mangrove forest Sundarban. Thus, these two routes are ecologically different from each other. Samples of these two routes have some genetic differences, because most of the locations (MK, CF and BL with PP and KN) of these two estuarine routes had significant *P* value, but their F_ST_ value was not satisfactorily high to make population differences. Ecological differences of these two routes might be played an important role to create this type of slight differences among them. Therefore, these scenarios were not significant enough to describe noteworthy differences in the population level, but may make a sign of upcoming population differences.

## Conclusion

In conclusion, the Hilsa shad collected from diverse habitats of Bangladesh belonged to the same population without mentionable more clusters. Although, recently Hilsa shad supply in Bangladesh is almost satisfactory but genetic diversity of this fish was very poor. Because of breeding failure of large group in the breeding migration and changing spawning pattern, the fish might experience a genetic bottleneck currently. This scenario is not a good sign for the survival of this population. Bangladesh, India and Myanmar already took some fisheries management strategy that may increase their number but failed to increase the genetic variation. Therefore, all three coastal countries of the Bay of Bengal should take a joint plan for the fisheries management and conservation of this fish species.

## Supplementary Information

Supplementary Information.

## Data Availability

Gene-capture data with adapters and low-quality reads were deposited in NCBI (PRJNA643346).
