# Supplementary material for: Cross-species gene enrichment revealed a single population of Hilsa shad (Tenualosa ilisha) with low genetic variation in Bangladesh waters
Source: Sci Rep. 2021 Jun 2;11:11560. doi: 10.1038/s41598-021-90864-6 (PMC8173019; doi:10.1038/s41598-021-90864-6)
Supplement: Supplementary file 1 — Supplementary Information. [file 41598_2021_90864_MOESM1_ESM.docx]

**Cross-species gene enrichment revealed a single population of Hilsa shad (*Tenualosa ilisha*) with low genetic variation in Bangladesh waters**

Anirban Sarker^1,2^, Junlong Jiang^1^, Habibon Naher^1,2^, Junman Huang^1^, Kishor Kumar Sarker^1^, Guoxing Yin^1^, Mohammad Abdul Baki^2^, and Chenhong Li^1*^

*^1^Shanghai Universities Key Laboratory of Marine Animal Taxonomy and Evolution, Shanghai Ocean University, Shanghai 201306, China*

^2^*Department of Zoology, Jagannath University, Dhaka-1100, Bangladesh*

* Author for Correspondence: Email: [*chli@shou.edu.cn*](mailto:chli@shou.edu.cn)

**Supplementary materials:**

**Table S1.** Taxon sampling, summary of the sequencing result of 139 individuals of *Tenualosa ilisha*  and 5 out-groups.

**Table S2.** Best K of structure analysis from delivered 14 K (1-14).

**Figure S1.** Plot of a DAPC analysis on 842 polymorphic SNP markers of Hilsa shad collected from 18 different locations. Each colored point represents a different sampling location.

**Figure S2.** Delta K summarized from the results of structure analysis.

**Figure S3.**  A ML tree (enlarged) based on sequences data concatenating 2,461 loci reconstructed by using IQtree v1.6.9 with 1,000 bootstrap replicates. Each color indicates specific habitat group and abbreviated letter indicates sampling location (Table 1).

**Figure S4.**  Dendrogram based on the F_ST_ values of populations of *T. ilisha* in the Bangladesh waters using the upgma method. Letters for sampling locations ( BG, KN, MK etc.) correspond to Figure 1.

| Voucher number | Collection site | No. raw reads | No. filtered reads | No. unique reads | No. captured loci |
| --- | --- | --- | --- | --- | --- |
| CM_CL_1364-1 | Chilmari (CM) | 13811166 | 13770380 | 12378356 | 2223 |
| CM_CL_1364-2 | Chilmari (CM) | 10128048 | 10096894 | 9139470 | 2204 |
| CM_CL_1364-3 | Chilmari (CM) | 8626140 | 8596176 | 7737418 | 2120 |
| CM_CL_1364-4 | Chilmari (CM) | 12706566 | 12664196 | 11515724 | 2215 |
| CM_CL_1364-5 | Chilmari (CM) | 5946254 | 5926902 | 5339712 | 2006 |
| CM_CL_1364-6 | Chilmari (CM) | 9108906 | 9084952 | 8189708 | 2128 |
| CM_CL_1364-7 | Chilmari (CM) | 8011662 | 7988546 | 7135530 | 2076 |
| CM_CL_1364-8 | Chilmari (CM) | 4260856 | 4246092 | 3758114 | 1848 |
| BG_CL_135O_1 | Balashi Ghat (BG) | 3249358 | 3233578 | 3158416 | 797 |
| BG_CL_135O_3 | Balashi Ghat (BG) | 2011698 | 2001866 | 1962534 | 580 |
| BG_CL_135O_4 | Balashi Ghat (BG) | 1715206 | 1706066 | 1672446 | 506 |
| BG_CL_135O_5 | Balashi Ghat (BG) | 2898068 | 2882040 | 2821348 | 717 |
| BG_CL_135O_9 | Balashi Ghat (BG) | 3434456 | 3417984 | 3349224 | 809 |
| BG_CL_135O_10 | Balashi Ghat (BG) | 3658620 | 3646380 | 3563636 | 865 |
| CN_CL_1348_1 | Chapai-Nababganj (CN) | 5460240 | 5433934 | 5173636 | 1576 |
| CN_CL_1348_2 | Chapai-Nababganj (CN) | 7422058 | 7393286 | 7126870 | 1866 |
| CN_CL_1348_3 | Chapai-Nababganj (CN) | 4946970 | 4919700 | 4744886 | 1298 |
| CN_CL_1348_4 | Chapai-Nababganj (CN) | 8158104 | 8112066 | 7829146 | 1788 |
| CN_CL_1348_5 | Chapai-Nababganj (CN) | 7477950 | 7442338 | 7093666 | 1826 |
| CN_CL_1348_6 | Chapai-Nababganj (CN) | 12182784 | 12116040 | 11768762 | 2055 |
| CN_CL_1348_7 | Chapai-Nababganj (CN) | 6998674 | 6972692 | 6671896 | 1820 |
| RS_CL_1349_1 | Rajshahi (RS) | 2593042 | 2579148 | 2523682 | 705 |
| RS_CL_1349_2 | Rajshahi (RS) | 3005774 | 2987772 | 2926326 | 854 |
| RS_CL_1349_3 | Rajshahi (RS) | 4578576 | 4557288 | 4451074 | 1100 |
| RS_CL_1349_4 | Rajshahi (RS) | 1545700 | 1539948 | 1490698 | 505 |
| RS_CL_1349_5 | Rajshahi (RS) | 3743828 | 3726174 | 3640282 | 930 |
| RS_CL_1349_6 | Rajshahi (RS) | 4847444 | 4824676 | 4716432 | 1136 |
| RS_CL_1349_7 | Rajshahi (RS) | 2637034 | 2623702 | 2551530 | 838 |
| RS_CL_1349_8 | Rajshahi (RS) | 3343432 | 3331830 | 3244150 | 933 |
| SS_CL_2318_1 | Someshwari, Durgapur (SS) | 8186078 | 8162586 | 7236474 | 1682 |
| MO_CL_2317_1 | Mohanganj (MO) | 4945970 | 4914700 | 4743886 | 1264 |
| MO_CL_2317_2 | Mohanganj (MO) | 1474100 | 1469150 | 1439132 | 1264 |
| MG_CL_1365_1 | Manikganj (MG) | 4662144 | 4630382 | 4444390 | 1458 |
| MG_CL_1365_2 | Manikganj (MG) | 3044048 | 3024420 | 2916418 | 1417 |
| MG_CL_1365_3 | Manikganj (MG) | 10784194 | 10717416 | 10436148 | 2021 |
| MG_CL_1365_4 | Manikganj (MG) | 3539284 | 3518634 | 3429402 | 1559 |
| MG_CL_1365_5 | Manikganj (MG) | 5156936 | 5120814 | 4963324 | 1582 |
| MG_CL_1365_6 | Manikganj (MG) | 5762632 | 5713332 | 5499352 | 1653 |
| MG_CL_1365_7 | Manikganj (MG) | 2985114 | 2969022 | 2895798 | 1467 |
| MG_CL_1365_8 | Manikganj (MG) | 3146242 | 3129286 | 3042912 | 1475 |
| MG_CL_1365_9 | Manikganj (MG) | 2893892 | 2866712 | 2820774 | 1261 |
| MG_CL_1365_10 | Manikganj (MG) | 4890614 | 4868742 | 4749830 | 1590 |
| BR_CL_1363_1 | Bhairab (BR) | 2237068 | 2230024 | 2160456 | 1463 |
| BR_CL_1363_2 | Bhairab (BR) | 3033758 | 3021946 | 2943490 | 1711 |
| BR_CL_1363_3 | Bhairab (BR) | 4653664 | 4637208 | 4525828 | 1796 |
| BR_CL_1363_4 | Bhairab (BR) | 1925434 | 1917890 | 1863894 | 1466 |
| BR_CL_1363_5 | Bhairab (BR) | 2425418 | 2412754 | 2358956 | 1435 |

| Voucher number | Collection site | No. raw reads | No. filtered reads | No. unique reads | No. captured loci |
| --- | --- | --- | --- | --- | --- |
| BR_CL_1363_6 | Bhairab (BR) | 2708144 | 2691764 | 2633914 | 1463 |
| BR_CL_1363_7 | Bhairab (BR) | 3203104 | 3191942 | 3114532 | 1693 |
| BR_CL_1363_8 | Bhairab (BR) | 2281852 | 2274384 | 2214580 | 1568 |
| BR_CL_1363_9 | Bhairab (BR) | 2929778 | 2918956 | 2844996 | 1572 |
| BR_CL_1363_10 | Bhairab (BR) | 1697898 | 1692930 | 1638692 | 1261 |
| CP_CL_2026_1 | Chandpur (CP) | 3913740 | 3904196 | 3626116 | 1237 |
| CP_CL_2026_2 | Chandpur (CP) | 5782332 | 5760684 | 5565320 | 1968 |
| CP_CL_2026_3 | Chandpur (CP) | 6495728 | 6474280 | 6239102 | 2003 |
| CP_CL_2026_4 | Chandpur (CP) | 6997098 | 6973130 | 6755444 | 2042 |
| CP_CL_2026_5 | Chandpur (CP) | 5796862 | 5781770 | 5582250 | 1947 |
| CP_CL_2026_6 | Chandpur (CP) | 8034724 | 8001192 | 7747976 | 2054 |
| CP_CL_2026_7 | Chandpur (CP) | 9924588 | 9895242 | 9555022 | 2120 |
| CP_CL_2026_8 | Chandpur (CP) | 1220266 | 1216046 | 1182070 | 1167 |
| CP_CL_2026_9 | Chandpur (CP) | 3667290 | 3656826 | 3546208 | 1629 |
| CP_CL_2026_10 | Chandpur (CP) | 8573376 | 8543496 | 8265878 | 2059 |
| KN_CL_2002_1 | Khulna (KN) | 2239440 | 2229232 | 2184006 | 770 |
| KN_CL_2002_2 | Khulna (KN) | 1187420 | 1178102 | 1160406 | 563 |
| KN_CL_2002_3 | Khulna (KN) | 2692208 | 2672564 | 2628558 | 917 |
| KN_CL_2002_4 | Khulna (KN) | 1768066 | 1758304 | 1725466 | 614 |
| KN_CL_2002_5 | Khulna (KN) | 1378056 | 1372314 | 1347520 | 504 |
| KN_CL_2002_6 | Khulna (KN) | 1558194 | 1544652 | 1522372 | 594 |
| KN_CL_2002_7 | Khulna (KN) | 1841244 | 1833856 | 1796790 | 642 |
| KN_CL_2002_8 | Khulna (KN) | 2010714 | 2001242 | 1966852 | 735 |
| PP_CL_1355_1 | Pirojpur (PP) | 4446360 | 4427974 | 4249026 | 1568 |
| PP_CL_1355_2 | Pirojpur (PP) | 2260550 | 2254860 | 2156026 | 1424 |
| PP_CL_1355_3 | Pirojpur (PP) | 2832064 | 2821758 | 2682394 | 1425 |
| PP_CL_1355_4 | Pirojpur (PP) | 3538378 | 3528658 | 3315882 | 1549 |
| PP_CL_1355_5 | Pirojpur (PP) | 1975152 | 1968926 | 1867884 | 1389 |
| PP_CL_1355_6 | Pirojpur (PP) | 3436470 | 3426066 | 3280536 | 1454 |
| PP_CL_1355_7 | Pirojpur (PP) | 2739144 | 2731694 | 2595078 | 1424 |
| PP_CL_1355_8 | Pirojpur (PP) | 1443510 | 1438334 | 1380138 | 1141 |
| PP_CL_1355_9 | Pirojpur (PP) | 1980056 | 1972604 | 1877500 | 1125 |
| BL_CL_2036_1 | Bhola (BL) | 9813152 | 9807212 | 7528734 | 867 |
| BL_CL_2036_2 | Bhola (BL) | 12585340 | 12580082 | 9832118 | 1550 |
| BL_CL_2036_3 | Bhola (BL) | 2833670 | 2819978 | 2744640 | 1552 |
| BL_CL_2036_4 | Bhola (BL) | 5801366 | 5782568 | 5618120 | 1859 |
| BL_CL_2036_5 | Bhola (BL) | 2097918 | 2090926 | 2039666 | 1454 |
| BL_CL_2036_6 | Bhola (BL) | 7692470 | 7664554 | 7456214 | 1963 |
| BL_CL_2036_7 | Bhola (BL) | 2935426 | 2924236 | 2850212 | 1619 |
| BL_CL_2036_8 | Bhola (BL) | 4060456 | 4047608 | 3943978 | 1821 |
| BL_CL_2036_9 | Bhola (BL) | 6450196 | 6432626 | 6262420 | 1900 |
| BL_CL_2036_10 | Bhola (BL) | 3697384 | 3682590 | 3575396 | 1660 |
| MP_CL_1356_1 | Mohipur (MP) | 1407318 | 1401536 | 1348842 | 1130 |
| MP_CL_1356_2 | Mohipur (MP) | 507538 | 505120 | 489158 | 801 |
| MP_CL_1356_3 | Mohipur (MP) | 2270012 | 2259446 | 2185978 | 1451 |
| MP_CL_1356_4 | Mohipur (MP) | 1642400 | 1632818 | 1583932 | 1296 |
| MP_CL_1356_5 | Mohipur (MP) | 1945316 | 1937448 | 1868466 | 1321 |
| MP_CL_1356_6 | Mohipur (MP) | 496292 | 493622 | 478444 | 720 |
| MP_CL_1356_7 | Mohipur (MP) | 1894274 | 1886010 | 1819420 | 1427 |
| MP_CL_1356_8 | Mohipur (MP) | 1046048 | 1041428 | 1006812 | 1128 |
| MP_CL_1356_9 | Mohipur (MP) | 1074274 | 1069138 | 1030424 | 1074 |

| Voucher number | Collection site | No. raw reads | No. filtered reads | No. unique reads | No. captured loci |
| --- | --- | --- | --- | --- | --- |
| CF_CL_1361_1 | Char Fasson (CF) | 3413278 | 3386882 | 3331840 | 1432 |
| CF_CL_1361_2 | Char Fasson (CF) | 2733738 | 2716490 | 2669010 | 1309 |
| CF_CL_1361_3 | Char Fasson (CF) | 3544142 | 3515262 | 3469008 | 1450 |
| CF_CL_1361_4 | Char Fasson (CF) | 2314308 | 2294618 | 2246672 | 1221 |
| CF_CL_1361_5 | Char Fasson (CF) | 2374252 | 2360006 | 2315864 | 1229 |
| CF_CL_1361_6 | Char Fasson (CF) | 1490532 | 1478274 | 1456748 | 996 |
| CF_CL_1361_7 | Char Fasson (CF) | 2929638 | 2916388 | 2848970 | 1430 |
| CF_CL_1361_8 | Char Fasson (CF) | 2639246 | 2626640 | 2576840 | 1316 |
| PC_CL_1359_1 | Pokhkhir Char (PC) | 3728842 | 3728842 | 3631076 | 1561 |
| PC_CL_1359_2 | Pokhkhir Char (PC) | 4085080 | 4070014 | 3971756 | 1645 |
| PC_CL_1359_3 | Pokhkhir Char (PC) | 1775194 | 1766130 | 1727720 | 1214 |
| PC_CL_1359_4 | Pokhkhir Char (PC) | 4525550 | 4510024 | 4412892 | 1547 |
| PC_CL_1359_5 | Pokhkhir Char (PC) | 2981454 | 2971902 | 2905800 | 1443 |
| PC_CL_1359_6 | Pokhkhir Char (PC) | 2846758 | 2833274 | 2771898 | 1394 |
| PC_CL_1359_7 | Pokhkhir Char (PC) | 4983784 | 4962680 | 4857614 | 1761 |
| PG_CL_1357_1 | Patharghata (PG) | 1412062 | 1404084 | 1379374 | 11175 |
| PG_CL_1357_2 | Patharghata (PG) | 672666 | 668460 | 656732 | 908 |
| PG_CL_1357_3 | Patharghata (PG) | 451486 | 448394 | 440932 | 769 |
| PG_CL_1357_4 | Patharghata (PG) | 1602334 | 1592832 | 1566568 | 1284 |
| PG_CL_1357_5 | Patharghata (PG) | 1593076 | 1586812 | 1551266 | 1335 |
| PG_CL_1357_6 | Patharghata (PG) | 2326576 | 2314410 | 2251116 | 1469 |
| PG_CL_1357_7 | Patharghata (PG) | 2580676 | 2569668 | 2507650 | 1486 |
| PG_CL_1357_8 | Patharghata (PG) | 1611752 | 1604648 | 1570796 | 1349 |
| PG_CL_1357_9 | Patharghata (PG) | 1474100 | 1469150 | 1439132 | 1270 |
| PG_CL_1357_10 | Patharghata (PG) | 4073446 | 4053640 | 3967330 | 1720 |
| CB_CL_2042_1 | Cox’s Bazar (CB) | 7489716 | 7467744 | 6947030 | 1554 |
| CB_CL_2042_2 | Cox’s Bazar (CB) | 3642294 | 3629000 | 3529660 | 1677 |
| CB_CL_2042_3 | Cox’s Bazar (CB) | 5711334 | 5694114 | 5546204 | 1815 |
| CB_CL_2042_4 | Cox’s Bazar (CB) | 3589514 | 3576498 | 3473870 | 1646 |
| CB_CL_2042_5 | Cox’s Bazar (CB) | 5112886 | 5089682 | 4953372 | 1758 |
| CB_CL_2042_7 | Cox’s Bazar (CB) | 2363436 | 2356138 | 2278718 | 1494 |
| CB_CL_2042_8 | Cox’s Bazar (CB) | 4340680 | 4324252 | 4197258 | 1687 |
| CB_CL_2042_9 | Cox’s Bazar (CB) | 6333154 | 6313694 | 6129810 | 1901 |
| CB_CL_2042_10 | Cox’s Bazar (CB) | 5323646 | 5310320 | 5166652 | 1731 |
| MK_CL_1360-2 | Maheshkhali (MK) | 2225932 | 2224932 | 1636310 | 939 |
| MK_CL_1360-3 | Maheshkhali (MK) | 3848084 | 3846434 | 2804746 | 957 |
| MK_CL_1360-4 | Maheshkhali (MK) | 1846242 | 1845834 | 1281676 | 666 |
| MK_CL_1360-5 | Maheshkhali (MK) | 2117768 | 2117292 | 1497366 | 712 |
| MK_CL_1360-6 | Maheshkhali (MK) | 3520342 | 3517148 | 2501302 | 1158 |
| MK_CL_1360-7 | Maheshkhali (MK) | 4682642 | 4680366 | 3209546 | 1131 |
| MK_CL_1360-9 | Maheshkhali (MK) | 1514966 | 1513388 | 1042256 | 601 |
| **Outgroup** | | | | | |
| CL_2312_1 | Out-group | 3905374 | 3887138 | 3795998 | 1104 |
| CL_2312_10 | Out-group | 4222386 | 4196846 | 4104824 | 1104 |
| CL_2312_2 | Out-group | 4192228 | 4171334 | 4070996 | 1104 |
| CL_2312_3 | Out-group | 4850020 | 4827432 | 4689222 | 1104 |
| CL_2312_6 | Out-group | 3918244 | 3896564 | 3818888 | 1104 |

**Table S1.** Taxon sampling, summary of the sequencing result of 139 individuals of *Tenualosa ilisha* and 5 out-groups.

**
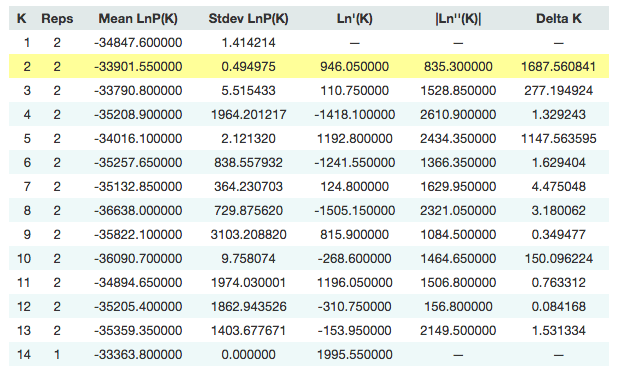
**

**Table S2.**  Best K of structure analysis from delivered 14 K (1-14).

**
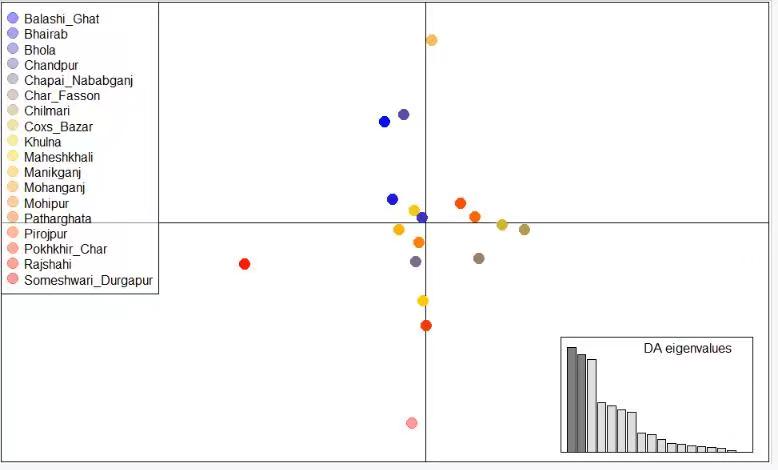
**

**Figure S1.** Plot of a DAPC analysis on 842 polymorphic SNP markers of Hilsa shad collected from 18 different locations. Each colored point represents a different sampling location.


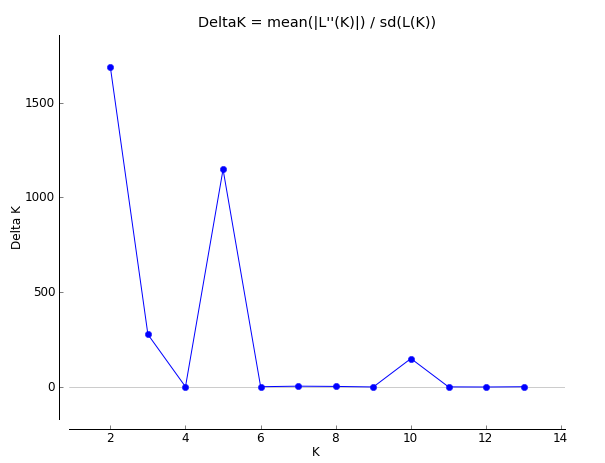


**Figure S2.** Delta K summarized from the results of structure analysis.


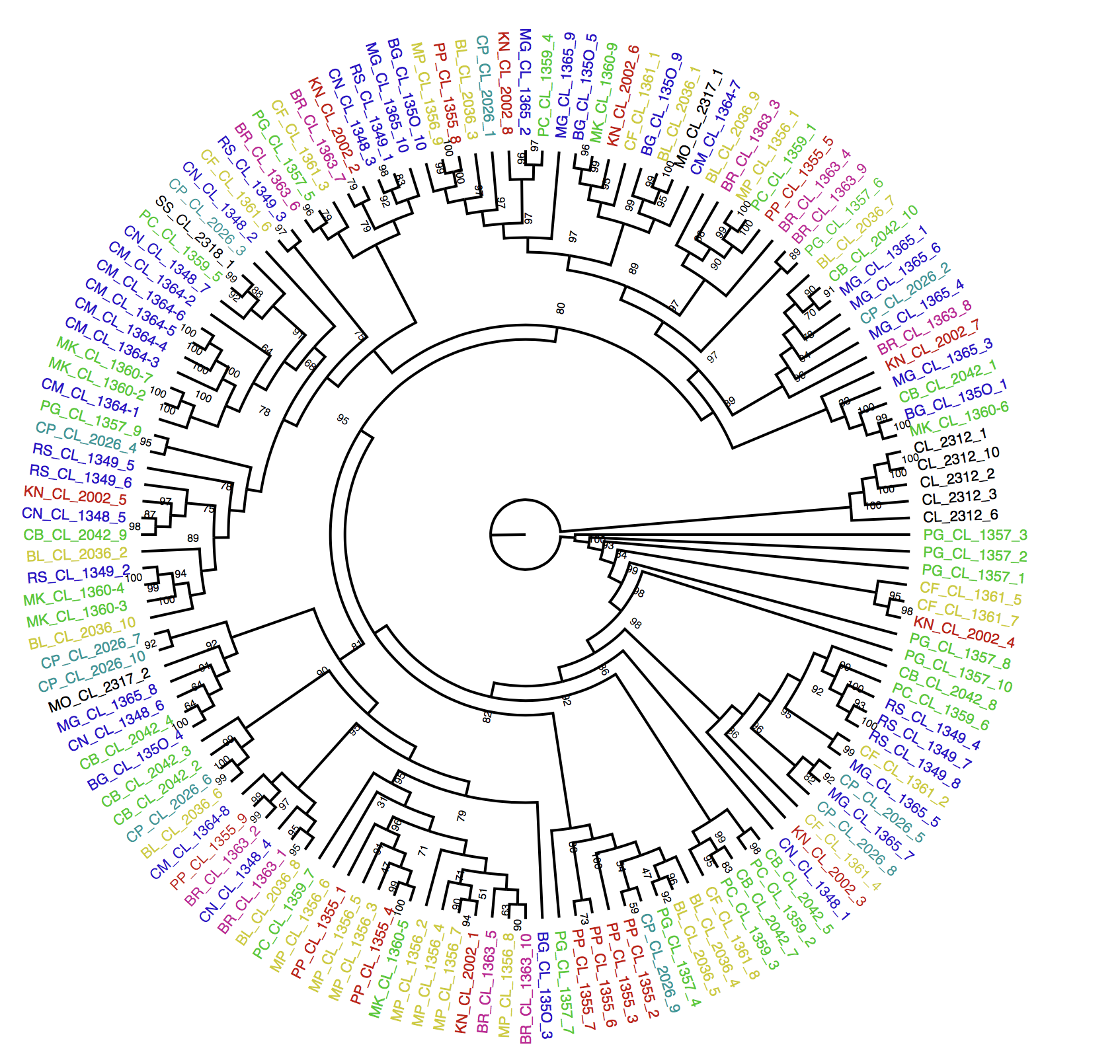


**Figure S3.**  A ML tree (enlarged) based on sequences data concatenating 2,461 loci reconstructed by using IQtree v1.6.9 with 1,000 bootstrap replicates. Each color indicates specific habitat group and abbreviated letter indicates sampling location (Table 1).


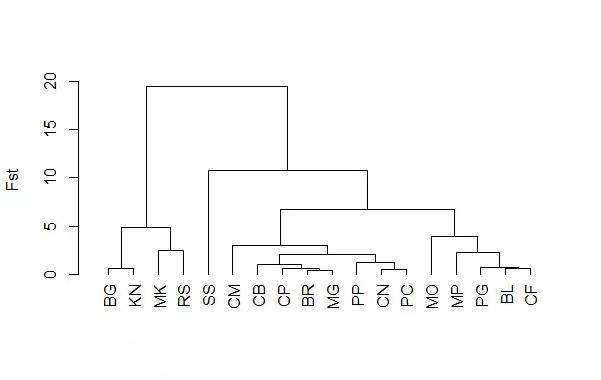


**Figure S4.**  Dendrogram based on the F_ST_ values of populations of *T. ilisha* in the Bangladesh waters using the upgma method. Letters for sampling locations ( BG, KN, MK etc.) correspond to Figure 1.
